# A Review of a Class of Emerging Contaminants: The Classification, Distribution, Intensity of Consumption, Synthesis Routes, Environmental Effects and Expectation of Pollution Abatement to Organophosphate Flame Retardants (OPFRs)

**DOI:** 10.3390/ijms20122874

**Published:** 2019-06-12

**Authors:** Jiawen Yang, Yuanyuan Zhao, Minghao Li, Meijin Du, Xixi Li, Yu Li

**Affiliations:** 1 College of Environmental Science and Engineering, North China Electric Power University, Beijing 102206, China; yangjiawen236@163.com (J.Y.); zyy950210@outlook.com (Y.Z.); limh8765@hotmail.com (M.L.); mjdu0401@outlook.com (M.D.); 2 The Moe Key Laboratory of Resources and Environmental Systems Optimization, North China Electric Power University, Beijing 102206, China; 3 Faculty of Engineering and Applied Science, Memorial University, St. John’s, NL A1B 3X5, Canada; xixi.li@mun.ca

**Keywords:** organophosphate flame retardants, distribution, environmental impacts, synthetic routes, pollution abatement

## Abstract

Organophosphate flame retardants (OPFRs) have been detected in various environmental matrices and have been identified as emerging contaminants (EC). Given the adverse influence of OPFRs, many researchers have focused on the absorption, bioaccumulation, metabolism, and internal exposure processes of OPFRs in animals and humans. This paper first reviews the evolution of various types of flame retardants (FRs) and the environmental pollution of OPFRs, the different absorption pathways of OPFRs by animals and humans (such as inhalation, ingestion, skin absorption and absorption), and then summarizes the environmental impacts of OPFRs, including their biological toxicity, bioaccumulation, persistence, migration, endocrine disruption and carcinogenicity. Based on limited available data and results, this study also summarizes the bioaccumulation and biomagnification potential of OPFRs in different types of biological and food nets. In addition, a new governance idea for the replacement of existing OPFRs from the source is proposed, seeking environmentally friendly alternatives to OPFRs in order to provide new ideas and theoretical guidance for the removal of OPFRs.

## 1. Introduction

Organophosphate flame retardants (OPFRs) are emerging flame retardants (FRs), in addition to alumina trihydrate (ATH) and halogenated flame retardants and nitrogen-based flame retardants, which can reduce the flammability of many consumer and building materials [1,2]. OPFRs are widely used in furniture, textiles, building materials, electronics and other processing chemicals and are one of the most commonly used FRs. OPFRs are also often used as plasticizers in floor polishes, coatings, engineering thermoplastics and epoxy resins [3].

OPFRs have a wide range of physical and physiological properties in the environment, for example, their solubility, log*K*_ow_ value, vapour pressure (VP), and bioconcentration factor (BCF) are absolutely unique. These properties are important factors in assessing the behaviour of OPFRs in the environment and their impact on organisms [4]. Volatile OPFRs with higher vapour pressures, such as tributylphosphat (TBP), triethyl phosphate (TEP) and tri(2-chloroethyl)phosphate (TCEP), tend to be more easily discharged into the air and deposited on dust than the larger/heavier OPFRs [5,6], while aryl and alkyl OPFRs with higher molecular weights are more hydrophobic and have similar BCFs and a greater affinity for sediments and soils; chlorinated OPFRs have been shown to have better water solubility and pose a continuing threat to aquatic animals [4,7].

Some brominated flame retardants (BFRs), such as polybrominated diphenyl ethers (PBDEs), have been phased out of the global FR market due to their increasing environmental persistence and evidence of bioaccumulation and toxicity. For example, pentabromodiphenyl ether (PentaBDE) and octabromodiphenyl ethers (OctaBDE) have been banned worldwide [2]. Because of these bans, other organic flame retardants, such as new brominated flame retardants (NBFRs) and OPFRs that are low in cost and have effective flame-retardant capabilities, have been used as substitutes for PBDEs in a variety of commercial products, since OPFRs are mostly used as replacements for the commercial PentaBDE mixture [8]. In 2011, 20% of the global FR production was attributed to BFRs and 15% was attributed to OPFRs. However, the safety of these alternative flame retardants (AFRs) compounds for humans and the environment must still be elucidated [9].

Compared with their predecessors, OPFRs have less persistence and biological enrichment, but in recent studies it has been found that due to their widespread usage, OPFRs are commonly detected in a variety of environmental matrices, including the atmosphere [10,11,12,13], dust [14,15], sediments [16], soil [17], arctic air [18], indoor air [2,19,20,21,22,23,24,25,26], ocean surface water [27], surface water [22,23,24,25,28], and a variety of biological samples [18,24]. Almost all of the OPFRs produced in the past decade have been detected in seawater and freshwater animals, poultry, insects and human samples. OPFRs have been identified as emerging pollutants.

OPFRs are used as additives by physical incorporation in the product without forming a chemical bond with the product matrix. Thus, it is easy for them to escape during the use of the product, resulting in OPFRs leaking into the environment via volatilization, wear, leaching, deposition, infiltration and dissolution [6,29]. In addition, humans are widely exposed to them through various routes such as skin contact, ingestion and inhalation. Non-intentional ingestion of dust particles and dermal absorption was indicated as a significant human exposure route for FRs [30,31].

Wastewater discharge from factories and wastewater treatment plants (WWTPs) and atmospheric deposition from industrial emissions are the most important pathways for OPFRs to enter the aquatic and terrestrial environment [32,33]. Water is the optimal distribution medium for OPFRs, thus OPFRs can be detected in surface water, sewage from the WWTPs, urban precipitation and storm water runoff. The concentration range of OPFRs in the above media is at a medium high level; they are some of the most important compounds detected in the water environment [29,34,35,36]. At present, the OPFRs TCEP, tris(2-butoxyethyl) phosphate (TBOEP) and tris(2-chloroisopropyl) phosphate (TCIPP) have been detected in the raw water and wastewater from potable water treatment plants [37,38].

There are potential sources of OPFRs, NBFRs, and PBDEs of the flame-retardant family in the indoor environment. For example, the levels of OPFRs are much higher than BFRs in family residences, schools, office space [5,39,40,41]. In these areas, the OPFRs are mainly found in building materials such as rigid polyurethane (PU) foam used in insulation, construction and refrigeration, flexible PU foam used in furniture and upholstery, electronic office equipment and electrical cable equipment; some representative polymers that incorporate OPFRs are acrylonitrile-butadiene-styrene (ABS), high impact polystyrene (HIPS), polyethylene, polyvinyl chloride (PVC), polypropylene, polyamides and some textiles [42,43], as well as certain baby products [44,45]. Halogenated OP triesters, tris(chloroisopropyl) phosphate (TCPP), tris(1,3-dichloroisopropyl) phosphate (TDCPP) and TCEP, are mainly used as FR in commercial products, including furniture, textiles, mattresses and electronics [4,46].

Most previous studies have focused on routinely detecting the existence of OPFRs in environmental media (oceans, lakes, rainfall and snow, WWTP influent and effluent, etc.) [33,47,48,49]; in contrast, relatively few studies have investigated OPFRs in biota, particularly in fish and wildlife, even though research and detection of OPFRs in organisms has been carried out during recent years.

It is estimated that by 2019 the annual production of global FR will reach six billion pounds, and phosphorus-based flame retardants are expected to account for 16% of the global market share [50,51]. In 1992, the total consumption of OPFRs worldwide was only 100,000 tons, while the consumption in 2011 was 500,000 tons and in 2015 it reached 680,000 tons [2]. Between 1995 and 2001, OPFR consumption in the global market increased from 108,000 tons to 186,000 tons [4,52]. According to market statistics for FRs, the global consumption of OPFRs has increased from 200,000 tons to 500,000 tons from 2004 to 2011 and is expected to be 680,000 tons in 2015 [4,14,53].

As more and more of these compounds are used, their environmental concentrations will further increase in the future [54,55]. Therefore, their impact on human health and the environment cannot be ignored. Although there has been increasing consensus on a wide range of OPFR exposures, it is worthwhile to ponder whether these compounds are actually safer than their predecessors [2,56].

Several adverse health consequences are associated with exposure to OPFRs, including neurotoxicity, developmental toxicity to humans and animals, damage to the reproductive function, endocrine disruption, and carcinogenicity [57]. TDCPP has been designated by the US Environmental Protection Agency (EPA) as a compound that has environmental persistence and is hazardous to human reproductive, genetic, and developmental functions [58].

Chlorinated OPFRs, such as TCEP, TCIPP and tris(1,3-dichloroisopropyl) phosphate (TDCIPP), have been shown to be neurotoxic and carcinogenic [59,60,61]. TDCIPP can easily enter the bloodstream, liver, kidneys, and testicles and can induce tumours [62]. It was found that TDCIPP levels in house dust were associated with decreased levels of male thyroid hormone (THs) and elevated levels of prolactin [31].

Some OPFRs have the potential to affect endocrine function, the central nervous system and reproductive system. Because of the endocrine disrupting effects of OPFRs on organisms and their health risks to neurodevelopment, liver and behavioural abnormalities, as well as the evidence that many of them may have carcinogenic properties, exposure to OPFRs is very worthy of attention [31,58,63].

It was widely found that in animal studies, OPFRs induce damaging changes in immunity, metabolism, genetics and endocrine activity. Studies of the bioaccumulation, metabolism, and toxicokinetics of OPFRs can be traced back to the 1970s [2,64,65,66,67]. In addition, OPFRs also have considerable long-range mobility. Some OPFRs such as TCEP, TBOEP, TCIPP, and TDCIPP were found to be present in air, water, and sediment samples collected throughout the northern hemisphere, and recently from the Arctic pole and East Antarctic ice sheet [2,18].

The use of FR can reduce fire-related deaths and injuries, and so it is for this reason that the residue of FRs is all over the world. While there are a growing number of scientists and engineers who are questioning how the statistics are collected and the assumptions used to fill the very large gaps in information, the risks and benefits have to be examined to determine the actual effects of FRs. There is concern about exposure to these substances due to the potential toxicity of certain FRs to the environment and human health.

At present, the research on OPFRs mainly focuses on their existence and detection in different media. A variety of accurate and sensitive detection methods have been developed that can accurately detect the concentration levels of OPFRs in various media, thus allowing a large number of animal experiments concerning the physiological toxicity of OPFRs. The toxicity mechanism has also gradually become clear. Considering the limitations of the previous single discussion on the scope of OPFRs research and the recent published research on OPFRs, this paper updates and comprehensively evaluates the information from these studies.

However, regarding the removal of OPFRs, there is no effective degradation or adsorption method for the complete elimination of OPFRs. This paper aims to propose a new idea for OPFRs removal in order to provide a theoretical reference for the removal of OPFRs.

## 2. Synthesis and Application of Flame Retardants

### 2.1. Usage Situation of Flame Retardant

FRs are widely used in various commodities as a functional additive to materials for the prevention of combustion, although FRs generally do not prevent combustion in most cases, especially when there is a large ignition source, however it is required to meet certain conditions [52]. The use of flame retardants dates back to 484 BC when the Greek historian Herodotus documented the use of potassium and aluminium sulphates by the Egyptians as flame retardants for soaking wood. In 1820, Gay-Lussac put forward the use of phosphoramide as a flame retardant for jute and linen weaving, thus laying the foundation for later research on nitrogen-containing compounds. In 1913, the chemist Perkin treated cotton linters with sodium stannate and ammonium sulphate, and tin oxide embedded in cotton linters was used as a flame retardant. During World War II, synthetic polymeric materials appeared. Traditional water-soluble flame retardants consisting of inorganic salts could not play the role of flame retardants on these hydrophobic synthetic polymer materials, and it became urgent to find novel flame retardants to meet new fire-resistance requirements for synthetic polymer materials [68,69]. The representative new flame retardants, chlorinated paraffin and antimony oxide, have been used as a composite flame retardant since 1930. In the 1950s, the invention of the reactive flame retardant chlorendic acid was rapidly extended to inventions of other reactive flame retardants containing phosphorus and halogens. As the above flame retardants could not meet the flame-retardant requirements for polyethylene plastic, in 1956 inert materials such as aluminium hydroxide began to be used as flame-retardant fillers for plastics [70,71,72]. In 1960, DechloranePlus (DCRP) was introduced as a cyclic compound flame retardant [73,74]. In the 1980s, halogenated phosphorus- and nitrogen-based additive-type flame-retardant polymers began to be widely used as a new type of flame retardant in a large number of commodities, in which bromine-based flame retardants were used as organic halogen-based flame retardants [2,3,4,75,76,77,78,79]. These were favoured by consumers due to their high flame-retardant efficiency and low production cost. They occupied a dominant position in the flame-retardant category and their consumption accounted for 85% of the total amount of organic flame retardants. With the recognition of organic pollutants, bromine-based flame retardants have been banned worldwide due to their environmental persistence, biotoxicity, bioaccumulation and migration characteristics since the 1990s [80,81]. OPFRs were put into use as early as the early 20th century, and their production and usage increased rapidly after 1940. However, they did not develop into mainstream flame retardants until the 1970s and 1980s, when bromine-based flame retardants gradually faded out in the flame-retardant market due to regulatory limitations on their production and use. Thus, OPFRs gradually developed into emerging mainstream products because of their excellent flame resistance and plasticizing effects when used as a substitute. The production of organophosphate ester flame retardants alone increased from 100,000 tons in 1992 to 341,000 tons in 2007 [52,53].

Most of the flame retardants listed in the Stockholm Convention (http:// www.pops.int/Home/tabid/2121/Default.aspx) that are banned by persistent organic pollutants (POPs) regulations are brominated flame retardants, of which most of those banned are the PBDE flame retardants. According to the EU’s “Registration, Evaluation, Authorization and Restriction of Chemicals” (REACH), some flame retardants are highly detectable substances and should not be found in commercial products at levels greater than 0.1% (Table 1).

The consumption of flame retardants in the United States in 2005 and 2008 was 65,000 tons and 72,000 tons, respectively, and for those years in in Europe were 83,000 tons and 95,000 tons, respectively. Global flame-retardant consumption mainly occurs in North America, Western Europe, Japan, parts of Asia (China, India, and South Korea), accounting for 34%, 29%, 10%, 27% of consumption, respectively [82]. In 2008, the global consumption of flame retardants was 1,950,000 tons, and in 2014 was 2,620,000 tons. The annual growth rate of global flame retardants is 4.9%, and the growth rate of demand in North America and Western Europe is slightly lower, with annual growth rates of 5.5% and 3.5%, respectively. The Asia Pacific region (China, India, Japan and South Korea) has the largest FRs regional market, accounting for 55% of global FR consumption in 2018 [54,61,83]. The growth of the flame-retardant market will exceed 7%, which is the main driving force for global flame-retardant growth [52,84,85]).

The global usage amounts of phosphorus-based flame retardants in 2001, 2007 and 2011 were 18,600 tons, 20,000 tons, and 29,200 tons, respectively. It can be seen that the global usage amount of phosphorus-based flame retardants increases year by year [7,35,54].

### 2.2. Classification and Preparation Technology of OPFRs

#### 2.2.1. Classification and Preparation Technology of Organophosphate Esters

Organophosphate esters are used in a multitude of applications such as flame retardants, plasticizers and lubricants. The organophosphate ester flame retardants are flame retardants that use phosphorus oxychloride as a main raw material and are prepared using compounds such as alcohols or phenol. These occupy the largest proportion of OPFRs.

The organophosphate ester flame retardants can be classified into three types according to their different ester groups: halogenated (alkyl-) OPFRs, alkyl (alkoxyl)- OPFRs, and aryl OPFRs. These also can be classified according to their different elements. There are organophosphate ester flame retardants containing only phosphorus, those also containing nitrogen and those also containing halogens. Among these, the nitrogen-containing organophosphate ester flame retardants have a better flame retardant efficiency than organophosphate ester flame retardants containing only phosphorus because of the synergistic effect between nitrogen and phosphorus [86].

Resorcinol bis(diphenyl phosphate) (RDP) is an early development of organic liquid flame retardants containing only phosphorus. It is widely used in engineering plastics, electrical and electronic products, etc. It is used as a substitute for TCEP, TCPP and decabromodiphenyl oxide (DBDPO) flame retardants because of its low volatility, meaning it is unlikely to be released into the environment [87,88]. However, these chemicals will probably be released over a longer period of time, especially at the end-of-life, thus we should also be cautious to the potential risk of emerging chemicals such as RDP.

**Figure 1:**
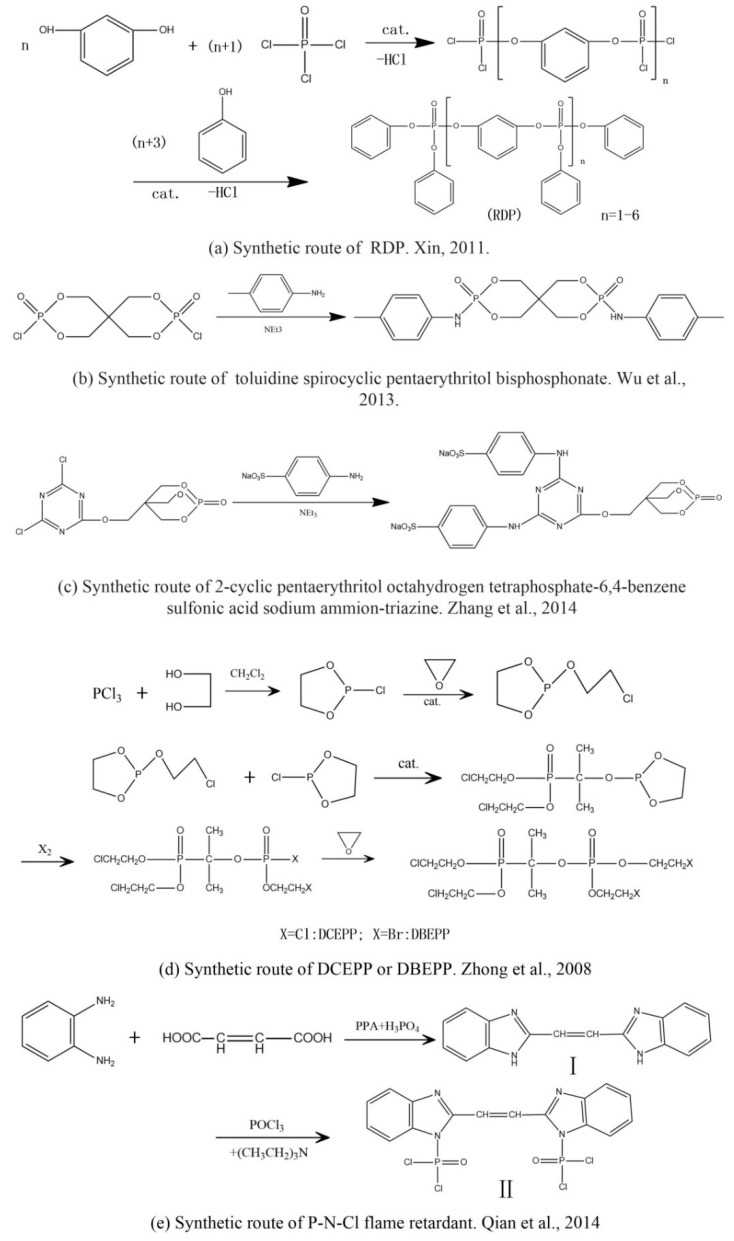
Synthetic routes of five Organophosphate flame retardants (OPFRs).

Xin’s research in 2011 showed that RDP can be preferentially synthesized by using resorcinol, phosphorus oxychloride and phenol as raw materials with MgCl_2_ as a catalyst [89]. In addition, after the thermal weight loss test, it was found that the mass loss was only 5% when the temperature was 315 °C, at which temperature the synthesis effect was the best. The synthetic route of RDP was shown in Figure 1a.

The nitrogen-containing organophosphate ester flame retardants contain both nitrogen and phosphorus, so their flame-retardant effects should be better than those of the compounds containing only phosphorus. In addition, their applications are more extensive and are currently attracting more attention. The flame retardant mechanism of the traditional phosphorus flame retardant is to form phosphoric acid to promote cationic crosslinking after combustion, thus forming a carbon layer to cover the polymer surface to prevent combustion, and on the basis of the above, when nitrogen is added to an organophosphate ester flame retardant, the nitrogen compound emits a large amount of non-toxic non-combustible gas after being heated. These gases can effectively block the supply of oxygen, which further achieves the purpose of flame retardancy and efficiency under the synergistic effect of nitrogen and phosphorus [90,91]. A novel monomolecular P-N intumescent flame retardant, toluidine spirocyclic pentaerythritol bisphosphonate was synthesized by nucleophilic substitution reaction with chlorospirophosphate and p-toluidine as raw materials. Acetonitrile was used as the solvent and triethylamine was used as acid binding agent. The acid binding agent was reacted at 80 °C for 4 h and the yield was as great as 79.3%. After testing, the initial decomposition temperature of the product was found to be 220 °C. When the temperature reached 500 °C, the char formation rate was 43.3% [92]. The synthetic route of toluidine spirocyclic pentaerythritol bisphosphonate is shown in Figure 1b.

Using 2-cyclic pentaerythritol octahydrogen tetraphosphate-4,6-dichlorotriazine and sodium sulfanilate as raw materials, a P-N intumescent flame-retardant 2-cyclic pentaerythritol octahydrogen tetraphosphate-6,4-benzene sulfonic acid sodium ammion-triazine integrating a carbon source, an acid source and a gas source was synthesized by a substitution reaction. The test results showed that the best effect of the new P-N intumescent flame retardant added to rigid polyurethane foam is that the limiting oxygen index of the flame retardant reached 27.5%, and the flame-retardant level reached the highest UL-V0 [93]. The synthetic route of 2-cyclic pentaerythritol octahydrogen tetraphosphate-6,4-benzene sulfonic acid sodium ammion-triazine is shown in Figure 1c.

Corrosive gases, carcinogens, and CO and HCN can be formed during the combustion process of halogen-containing organophosphate ester flame retardants but there are few reports on these products. However, due to its non-negligible flame-retardant efficiency, there are still some reports of the organophosphate ester flame retardants containing both chlorine and bromine, containing fluorine, and organophosphate esters flame retardant with a high halogen content. Zhong et al. synthesized two new halogen-containing organophosphate ester flame retardants, O,O′-di (2-chloroethyl),O″-[2-bis-(2-chloroethoxy) phosphoryl] propylphosphate (DCEPP) and O,O′-di (2-bromoethyl),O″-[2-bis-(2-bromoethoxy) phosphoryl] propylphosphate (DBEPP) [94]. The first synthesis of the intermediate 2-chloro-1,3,2-dioxapholane was carried out using phosphorus trichloride and ethylene glycol as raw materials, and then novel chlorine-containing organophosphate ester flame retardant DCEPP was synthesized by using ethylene oxide, acetone and a halogen such as chlorine or bromine as raw materials. The synthetic route of DCEPP or DBEPP is shown in Figure 1d.

Qian et al. synthesized a novel benzimidazole type flame retardant with the synergistic effect of P-N-Cl with o-phenylenediamine, maleic acid and phosphorus trichloride as raw materials and triethylamine as a catalyst [95]. The synthesis principle of intermediate (I) and P-N-Cl flame retardant (II) is shown in Figure 1e.

The results showed that the flame-retardant yield was 73.6% under the reaction conditions of 75 °C for 8 h. By measurement, when the mass fraction of the P-N-C1 halogen-containing organophosphate ester flame retardant in unsaturated resin and epoxy resin is 12%, the limiting oxygen index (LOI) value of the flame retardants can reach 33.7% and 32.3%, respectively. Compared with the most widely used plastic flame retardant for ABS, for which the average LOI value is 26.8% [96], the P-N-C1 halogen-containing flame retardant has good flame-retardant properties and potential application prospects.

Most of the organophosphate ester flame retardants are liquid, have poor heat resistance, high volatility, inconvenient processing and poor compatibility with polymer materials. They constitute one of the problems that require further research in the future.

#### 2.2.2. Phosphonate Flame Retardant

Organic phosphonate flame retardants have properties similar to those of organophosphate ester flame retardants, including stable molecular properties and good water resistance and solvent resistance. Thus, they are promising flame retardants.

Nguyen and Kim obtained the intermediate phenyl methyl phosphonochloridate (PMPC) by reacting methyl phosphonic dichloride (MPDC) with phenol, followed by reacting PMPC with piperazine to obtain the final product diphenyl piperazine-1,4-diylbis(methylphosphinate) (DPPMP) with high purity [97]. The flame retardancy of the polymer with the addition of flame retardant DPPMP could reach UL-94 V-0, under which the flame retardant DPPMP can increase the flame retardancy of the polymer and shows a good flame-retardant effect.

In 2015, a novel flame retardant, imidazole spirocyclic phosphoramidate (IPSA), was synthesized using spiralphosphodicholor (SPDPC) and imidazole monomer [98]. After the flame retardant was added to the cotton fabric, the LOI value of the flame-retardant cotton fabric was increased by 36.6% and the acceptable tensile strength was reduced by 13.3%; thus, the product has an excellent flame-retardant property.

#### 2.2.3. Phosphine Oxide Flame Retardant

Phosphine oxide has good hydrolytic stability and can be used as a flame retardant for aliphatic polyester-like macromolecular materials and is widely used because of its long-lasting flame retardancy.

In 2010, a polysulfone (PSF)-bismaleimide (BMI) modified epoxy matrix system, which has an intercross-linked network, was synthesized by using the diglycidyl ether of bisphenol A (DGEBA) epoxy resin, hydroxyl-terminated polysulfone and bismaleimide (3,30-bis(maleimidophenyl) phenylphosphine oxide) as reactant and diamino diphenylmethane as curing agent. The experiments by Rajasekaran et al. [99]. showed that the LOI value of modified epoxy resin increases with the increase in BMI dosage, and the introduction of new substances can significantly promote the flame retardancy of epoxy resin.

Huang et al. used 4,4′ diaminodiphenylsulfone, 2-hydroxybenzaldehyde and diphenylphosphine oxide to synthesize phosphorus/nitrogen-containing diphenylphosphine oxide derivatives, which besides being flame retardants, are also added to the epoxy resin as a curing agent copolymerized with the diglycidyl ether of bisphenol A [100]. The results showed that the new epoxy resin samples exhibit an excellent thermal stability and flame retardancy, with an LOI of 32.4% and a flame-retardant grade up to that of UL-94 V-0.

#### 2.2.4. Organophosphate Heterocycle Flame Retardants

Heterocycles are an important area of recent research on flame retardant and organophosphate chemistry. The organophosphate heterocycles mainly consist of five-membered rings, six-membered rings and spiro compounds, which can be used for flame retardant treatment of various materials such as polyester, epoxy resin and polyurethane. Furthermore, they can also function as plasticizers, heat stabilizers and flame retardants in the material.

Zhang et al. synthesized the star-shaped heterocyclic flame retardant hexakis (4-nitrophenoxy) cyclotriphosphazene (HNCP) containing P and N [101]. Applying HNCP to poly(ethylene terephthalate) (PET), the flame retardant grade UL-94 V-0 can be achieved when the added mass fraction of HNCP is 5%. During the combustion process, the flame retardant is preferentially decomposed, and a carbon layer having a continuous internal porous structure on the surface of the material is formed, which hinders the further decomposition of the polymer, thereby achieving a good flame-retardant effect.

In 2017, the polyphosphate poly (6-oxido-6H-dibenzo [-c,e] [1,2]oxa-phosphinin-6-yl phenyl phenylphosphate (POBPP) was synthesized by using 10-(2,5-dihydroxyl phenyl)-9,10-dihydro-9-oxa-10-phosphaphenanthrene-10-oxide (DOPO-BQ) and phenyl dichlorophosphate as reactants [102]. In addition, DGEBA epoxy resin cured with POBPP can pass the UL-94 V0 grade using a lower addition amount of 5 wt% and has excellent flame retardancy.

### 2.3. The Comparison of Several Common Flame Retardants

In various flame retardants, different compositions may contain halogens (bromine and chlorine), phosphorus, nitrogen, metals and minerals based on aluminium and magnesium. References [4,72], summarized that there were four major groups of flame retardants, including inorganic, halogenated organic, organophosphate and nitrogen-based flame retardants. The US Consumer Products Safety Commission (CPSC) commissioned the US National Academies to study whether classes of organohalogen FRs could be considered, and pointed out that, in the current age, considering the classes of organohalogen FRs, instead of studying and regulating each chemical separately, is the trend of the times for researchers.

Aluminium hydroxide is an inorganic flame retardant that is one of the most widely known and commonly used mineral flame retardants. As the temperature rises, Al(OH)_3_ undergoes an endothermic decomposition (1.17 kJ/g) at approximately 220 °C and absorbs energy. ATH shows its flame-retardant effect mainly by endothermic decomposition, diluting the combustible gases and forming an insulating protective layer. A brominated monomer such as brominated styrene or brominated butadiene is used in the production of brominated polymers, which are then blended with nonhalogenated polymers or introduced into the feed mixture prior to polymerization, resulting in a polymer containing both brominated and nonbrominated monomers. PBDEs are additive flame retardants and are the next highest production group of BFRs currently in use. PBDEs are produced by bromination of diphenyl ether in the presence of a Friedel–Craft catalyst (i.e., AlCl_3_) in a solvent such as dibromomethane, and toxic gases are also produced [72].

However, these halogenated compounds produce dioxin, furan, and other carcinogenic substances in the combustion process, which highly limits their applications in engineering; in addition, they are highly persistent in the environment [37,103,104,105]. To avoid the disadvantages of the halogen-containing flame retardants mentioned above, researchers have been actively pursuing new flame-retardant technology which mainly includes phosphorus, nitrogen, silicon, boron, zinc, iron, and aluminium-containing flame retardants. Among these, phosphorus-containing flame retardants, one category of the most promising halogen-free flame retardants, exhibit favourable flame retardant efficiency. The reason lies in that phosphorus containing compounds can undergo dehydration and carbonization to form protective carbon layers, thereby effectively reducing the flammability of the polymers [106,107,108,109]. The comparison of OPFRs with other FRs in physical and chemical t characteristics is shown in Table 2 [2,4,16,63,70,81,110,111].

**Figure 2:**
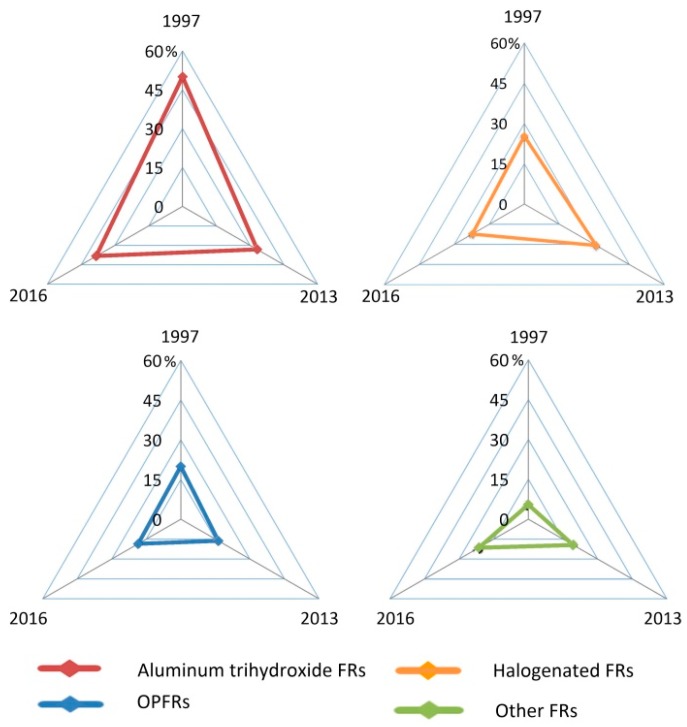
Sketch diagram of consumption of several main flame retardants in different years.

The consumption of flame retardants mentioned above (inorganic, halogenated organic, organophosphate and nitrogen-based flame retardants) accounted for 50%, 25%, 20% and >5% of the annual production, respectively, in 1997. In 2016, the most important flame-retardant product types by volume were aluminium trihydroxide, with 38% of the total market, followed by organophosphate compounds (18%), brominated compounds (17%), and antimony oxides (8%). The total consumption of FRs in 2006 was 465,000 tons, and the consumption of OPFRs was 85,000 tons in 2005 in Europe [53,112]. Approximately 311,000 metric tons of BFRs in 2005, and 500,000 tons of OPFRs in 2011 and estimated 680,000 tons of OPFRs in 2015 were used worldwide [4,14,113]. The above data are shown in Figure 2.

## 3. Detection of Organophosphate Flame Retardants

### 3.1. Traditional Method for Detecting Organophosphate Flame Retardants

Organophosphate flame retardants are widely used in materials encountered in daily living such as textiles, furniture, carpets, electronics and building insulation, and they are released in large quantities during the process of use, disposal and recycling [114,115]. Therefore, such flame retardants are ubiquitous in non-biological and biological environments [116,117]. Liquid chromatography-mass spectrometry (LC/MS) is the most traditional method for detecting organophosphate flame-retardant residues in the environment [118,119,120,121,122,123]. The method first requires all samples to be tested to be stored at a temperature of 5 ± 3 °C in a glass container. When the temperature returns to ambient temperature, the detection and analysis can be conducted [124]. Isotopic labels (TBP, d12-TCEP, d15-TDCPP, 13C18-TPHP (triphenyl phosphate), d10-BDCP (Tris-(2,4,6-Tribromophenoxy)dichlorophosphorane) and 13C2-TBEP (tris(2-butoxyethyl) phosphate)) are added to the glass container before instrumental analysis to monitor the performance of the method and the changes in the instrument [125]. In all, 0.1 mL of the standard solution and 0.1 mL of the ILIS spike solution are added to a 1.8 mL amber glass HPLC vial already containing 0.8 mL of high-performance liquid chromatography (HPLC) water [126]. Finally, 0.1 mL of ILIS spike solution and 0.1 mL of methanol are added to each HPLC vial [127]. In this way, each standard sample contains 20% methanol in aqueous solution, which meets ILIS levels and is ready for instrumental analysis [128].

Liquid chromatography-mass spectrometry avoids the time-consuming steps of sample concentration/enrichment detection. Its advantages include ease of operation, rapid detection and reliable results. This not only improves the efficiency of the instrumental analysis but also eliminates common pollution source of target analytes. Santin achieved simultaneous determination of 16 OPFRs in fish by liquid chromatography-quadrupole-linear ion trap mass spectrometry (LC-MS-MS) [125].

### 3.2. Emerging Methods for Detecting Organophosphate Flame Retardants

To further study the distribution of organophosphate flame retardants in the environment, scholars from various countries have developed some new detection methods based on LC/MS, such as liquid chromatography-tandem quadrupole mass spectrometry, microwave-assisted extraction combined with gel permeation chromatography and silica gel clean-up followed by gas chromatography–mass spectrometry [129], ion-pair liquid chromatography–tandem mass spectrometry [130], and liquid chromatography–electrospray ionization (+)– tandem mass spectrometry (LC-ESI/MS/MS) [131], etc., among which LC-ESI/MS/MS and microwave-assisted extraction combined with gel permeation chromatography and silica gel clean-up followed by gas chromatography–mass spectrometry are the most widely used methods.

#### 3.2.1. LC-ESI (+)-/MS/MS Method

Based on the traditional detection method for organophosphate flame retardants, Chen et al. developed a new quantitative analysis method [131]. It is based on a simple two-step sample extraction followed by liquid chromatography–electrospray ionization (+)– tandem mass spectrometry, which is a more sensitive method for detecting the amount of flame retardant in the environment. In this study, Chen et al. used this method to successfully detect the presence of seven halogen-free OPFRs, three chlorinated OPFRs, and two brominated OPFRs in silver gull eggs. Separation and quantification of target OPFRs was first performed on a Waters Quattro Ultima^®^ tandem quadrupole mass spectrometer (MS/MS) coupled with a Waters 2695 high-performance liquid chromatograph (HPLC) [132]. The LC system was equipped with a Waters Xterra^®^ Phenyl column (2.1 mm × 100 mm, 3.5-μm diameter) and the column temperature was kept at 40 °C. The mobile phases consisted of water (A) and methanol (B), both spiked with 0.1% formic acid (*v*/*v*) [15]. The MS system is equipped with electrospray ionization (ESI) probe operating in positive mode, using high purity nitrogen and argon nebulizing and collision gases, respectively. [49]. Detection and quantification of OPFR analysis was performed in a selected reaction monitoring (SRM) mode [133]. The other operation parameters for MS were as follows: capillary voltage: 4.0 kV; source temperature: 100 °C; probe temperature: 300 °C; cone gas flow: 150 L/h; desolvation gas flow: 700 L/h.

The above method has the following advantages: (1) simplicity and speed: since the removal of large volumes of lipid by GPC is not required, time and solvent consumption are greatly reduced; (2) sensitivity and accuracy: the LC-ESI (+)–/MS/MS instrument showed excellent sensitivity throughout the test. In addition to the accuracy of the instrumental analysis, good recovery, minimal matrix effects, and negligible background contamination together ensure the accuracy of the test data.

#### 3.2.2. Microwave-Assisted Extraction Combined with Gel Permeation Chromatography and Silica Gel Clean-Up Followed by Gas Chromatography–Mass Spectrometry

It is well known that lipid compounds have a significant impact on the performance of the column and the ion source of the mass spectrometer. In order to avoid the influence of these lipid compounds and other co-extracted interferences on the experimental results, Ma et al. used microwave-assisted extraction combined with gel permeation chromatography and silica gel clean-up followed by gas chromatography–mass spectrometry to measure 14 organophosphate flame retardants (halogenated OPFRs, non-halogenated OPFRs and triphenylphosphine oxide) in biological samples [129]. Before using microwave-assisted extraction combined with gel permeation chromatography and silica gel clean-up followed by gas chromatography–mass spectrometry to conduct instrumental analysis, it is required that the selected biological sample be cleaned to remove inedible parts to obtain a clean muscle sample, ground and crushed, and stored at −20 °C until experimental analysis [134]. Subsequently, the biological sample was extracted using the microwave assisted extraction (MAE) technique in the MarsX system (CEM, Matthews, NC, USA). Prior to this step, the extraction bottle was rinsed with HEX and the solvent was extracted. This process was carried out in a dark environment and used the ramp-to-temperature mode (ramp time: 10 min). To remove potential interference from lipids and other substances, the method uses GPC (300 mm × 300 mm i.d.) and SPE two-step removal techniques to eliminate interference from biological samples. Subsequent GC/MS analysis was then performed. Compared with the traditional method, this method has certain efficiency and the cleaning method is more comprehensive.

### 3.3. Detection of Organophosphate Flame-Retardant Content in Different Media

OPFRs can be transported over long distances by air and water, or accumulated in indoor environments [2,50], which results in extensive detection of OPFRs in air, water, soil, indoor environments and human food sources. In addition, in a study of 75 common foods in China including samples of foods such as dairy products, cereals, meat and agricultural products, the probability of detecting OPFR was 100%. In addition, the study also revealed that rice is the main potential source of human OPFR exposure [135].

#### 3.3.1. Detection of Organophosphate Flame-Retardant Content in Biological Samples

Organophosphate esters (OPEs) were found in human blood, milk, and placenta, and in fish and their eggs, which means OPFRs are very likely to be transferred to the next generation [3,131].

In 2014, Daryl et al. detected the presence of organophosphate flame retardants in *Salvelinus namaycush* collected from Canadian lakes, with TBOEP having the highest quantitative frequency, at an average concentration of 4.935 ng/g (ww) [47]. In 2017, Giulivo et al. found OPFRs in 27 species of fish samples from three European basins (the Evrotas, Greece; the Adige, Italy; and the Sava, Slovenia), in addition to Croatia, Bosnia and Herzegovina and Serbia [136]. The analysis showed that the presence of OPFRs was detected in all samples. In the Evrotas basin, the content of TBP in fish samples was the highest at 32.5 ng/g lw, followed by TCEP and tris(isopropylphenyl)phosphate (IPPP), 18.2 ng/g (lw) and 7.81 ng/g (lw), respectively. In the Adige basin, the highest TBP content was 102 ng/g (lw), followed by isodecyldiphenyl phosphate (IDPP) and ethylhexyl diphenyl phosphate (EHDP) at concentrations of 52.5 ng/g (lw) and 31.8 ng/g (lw), respectively. The fish samples collected in the Sava basin had the highest IPPP content, with an average concentration of 39.5 ng/g (lw), followed by TBP (average of 25.7 ng/g (lw)) and TCEP (average of 18.0 ng/g (lw)). It can be seen that although the three river basins have different modes, TBP, TCEP and IPPP are the most abundant organophosphate flame retardants in European fish.

Santín tested 16 target OPFRs in 12 river fish samples, 13 of which were detected in the tested samples [125]. The ΣOPFR was as high as 2423 ng/g lw, especially the content of IPPP, up to 601 ng/g lw. The three most frequently detected OPFRs were TBOEP (92%), tri(2-ethyltexyl)phosphate (TEHP) (83%) and IPPP (67%), which was the first report of IPPP and IDPP in biological samples. Santín also determined the levels of sixteen OPFRs in twelve river fish samples by a new method of liquid chromatography-tandem mass spectrometry from a Spanish River basin. Three OPFRs were detected most frequently, 92%, 83% and 67% for TBOEP, TEHP and IPPP, respectively. This was the first time that IDPP and IPPP were detected in biota samples.

Ma et al. found that tri-n-butyl phosphate (TnBP), TCEP, TCIPP and TBOEP were present in fish and bird muscle samples collected in Guangdong, China, with concentrations reaching as high as 4692 ng/g lw [129]. Additionally, McGoldrick et al. reported on OPFRs in *Salvelinus namaycush* collected in 2009–2010 from the aquatic environment across Canada including from Lakes Erie and Ontario [47]. Similar to findings in herring gull, TCEP and TBOEP were the most frequently quantified OPFRs and at similarly low concentrations.

Whole-body homogenates of lake trout or walleye collected from Canadian lakes were screened for OPFRs. TCEP, TBOEP were most frequently quantified with concentrations ranging from <0.07 to 9.8 ng/g (ww). The levels of TBOEP were highest in fish from the Great Lakes region while TCEP was detected only in fish from the northernmost lakes in the research network. Chen et al. also showed that five OPFRs were frequently quantifiable (including TPHP and TDCIPP) in individual herring gull eggs (*Larus argentatus*) collected from Lake Huron, and where TCIPP (0.21e4.1 ng/g ww), TCEP (0.02e0.55 ng/g ww), and TBOEP (0.16e2.2 ng/g ww) were most the concentrated of all OPFRs detected [131].

It can be seen that the most commonly detected OPFRs in organisms included TCEP, TBOEP and TCIPP, etc. As the detection technique improves and detection sensitivity increases, more OPFRs are detected in the living body.

#### 3.3.2. Detection of Organophosphate Flame-Retardant Content in Surface and Drinking Water

Contamination by OPFRs is an emerging concern in aquatic environments. In 2014, Hu et al. collected 13 seawater samples from the Yellow Sea and the East China Sea from Qingdao, Lianyungang, Xiamen and other places in China, and analysed the halogenated organophosphate flame retardants [137]. The test results showed that the total concentration of organophosphate flame retardants in the sample was 741.94 ng/L, and the TCEP, TCPP, TDCPP and tris(2,3-dibromopropyl) phosphate (TDBPP) concentrations were 134.44, 84.12, 109.28 and 96.70 ng/L respectively.

In the same year, Li et al. conducted a comprehensive survey of nine organophosphate flame retardants in drinking water in China [36]. The results revealed that the total concentration of OPFRs in tap water was 205.05 ng/L, of which TBEP, TPHP and TCPP are the most common components. In 2016, Khan et al. investigated the concentration of organophosphate flame retardants in drinking water in industrial, rural and background areas of Pakistan, and found that the total concentration of OPFRs in the above regional samples was 71.05 ng/L, of which the TCPP content was the most prominent [8].

In 2018, Zha et al. detected the existence of OPFRs in surface waters of the Changjiang River in China and conducted a quantitative analysis of OPFRs in surface waters using a modified polar organic chemical integrated sampler (m-POCIS) [138]. The results showed that the average concentration of OPFR was 47.04 ng/L, of which the highest concentration of TCPP was 16.51 ng/L. In the same year, Jakob et al. screened a series of residual OPFRs from 23 river water samples covering the entire latitude range in Sweden. In general, the average content of OPFRs was 56 ng/L [139].

Cristale et al. tested the presence and distribution of ten OPFRs in three Spanish rivers (Nalón, Arga, Besòs) [32]. The results indicated that OPFRs constitute a ubiquitous contaminant in water (the ΣOPFRs ranges from 0.0076 to 7.2 μ/gL). In addition, concentrations of Σ10 OPFRs in sediments from the Elbe River in Germany and three rivers (Nalón, Arga, Besòs) in Spain (ΣOPFRs ranging 3.8 to 824 μg kg^−1^) are much higher than those from Lake Huron of The Great Lakes by Cao et al. [140]. The highest concentrations of OPFRs were found in creeks taken near industrial complexes, and OPFR concentrations decreased with increasing distance from creeks to inshore or offshore lake water [52].

#### 3.3.3. Detection of Organophosphate Flame-Retardant Content in Sewage and Wastewater

Marklund et al. analysed effluents from seven Swedish sewage treatment plants. TBEP, TCPP and TBP were the most abundant compounds with concentrations ranging between 3100 and 11,000 ng/L, 1500 and 24,000 ng/L and 360 and 6100 ng/L, respectively [35]. The effluents of eight municipal WWTPs in Western Europe were analysed by Reemtsma et al. for the occurrence of 36 polar pollutants including two halogenated flame retardants, TCPP and TCEP [141]. Elena Martínezcarballo et al. investigated the content of OPFRs in 16 municipal sewage treatment plants in Austria [118]. There were basically seven OPE FRs (TEP, TCEP, TCPP, TPHP, tris(1,3-dichloropropyl)phosphate (TDCP), TBP and TBEP) in all wastewater samples. TCEP, TPHP, TBP had the lowest instrumental detection concentration, which were lower than the method quantification limits (MQL), while TBEP has the highest instrumental detection concentration of 5400 ng/L. The highest mean and maximum concentration levels were determined for TBEP and TCPP, whereas TPHP showed the lowest values.

On-going OPFR contamination by WWTP discharge was evidenced by the clear decrease in OPFRs in water samples collected moving away from WWTP outfalls. Additionally, TBEP and TEP were dominant in water samples [52]. This tendency agrees with previous investigations of OPFRs in wastewater.

#### 3.3.4. Detection of Organophosphate Flame-Retardant Content in Soil

In 2017, Hu et al. collected 48 surface sediments from three mangrove wetlands in the Pearl River Estuary (PRE) and studied the distribution of OPFRs [85]. The results showed that the average concentration of OPFRS in mangrove sediments was 195.15 ng/g, the composition of which is mainly TCPP (35.0%), followed by TCEP (19.0%), TBOEP (17.7%) and TEHP (10.1%). By 2018, Wang et al. determined the distribution of 12 OPFRs in the soil of a waste recycling area. It was found that the average concentration was 1079.25 ng/g, among which TCIPP was the main component in soil samples (average concentration is 54.7 ng/g), which is about one order of magnitude higher than other OPFRs. In addition, the concentration of TCEP and TDCIPP is also higher than other OPFRs, which are 9.53 and 8.35 ng/g, respectively [64]. In the same year, testing showed the average environmental concentrations of eight OPFRs in the topsoil of four major cities in Nepal to be 1,3962.5 ng/g [142]. From the compositional pattern, tris(methylphenyl) phosphate (TMPP) is the most abundant phosphorus chemical in the soil, followed by TCIPP, which accounts for 42% and 16.5% of the total flame retardants, respectively.

#### 3.3.5. Detection of Organophosphate Flame-Retardant Content in Sediment

Concentrations of 14 OPEs were measured in sediment core surface Ponar grab samples collected from Lakes Ontario, Michigan, and Superior of North America. The sum of the 14 OPEs in Ponar grab samples averaged 2.2, 4.7, and 16.6 ng/g dw in Lakes Superior, Michigan, and Ontario, respectively [140]. The main monomers of OPEs in the surface sediments of the Great Lakes are TBEP, tricresyl phosphate (TCrP) and TPHP, approximately a quarter (17 tons) of the total OPE burden (63 tons) in Lake Michigan resides in sediment, which may act as a secondary source releasing OPEs to the water column for years to come. Therefore, research on OPFRs in sediments is mandatory for environmental needs. The increase in OPEs in Lake Michigan sediments is mainly due to the chlorine-containing OPEs, especially TCPP. Its concentration has doubled in the past 20 years, becoming the most abundant and fastest growing OPFR, and the concentration (two relatively hydrophilic OPEs including TCPP) in the sediment is much higher than estimated based on the equilibrium between water and sediment organic carbon. In addition, multiple linear regression analysis showed a statistically significant correlation between the logarithmic concentration of Σ14OPEs and the distance factor from newly developed urban areas, which means that the concentration of OPEs was affected by urban pollution source emissions and human activities.

Cao et al. detected the presence of seven OPFRs (TBP, TBEP, TCEP, TCPP, TDCPP, TPHP, tritolyl phosphate (TTP)) in Tai Lake sediment by ultrasonic extraction and gas chromatography-mass spectrometry [16]. The concentration of Σ7OPs ranged from 3.38 to 14.25 µg/kg. TCPP, TCEP, and TBEP were the dominant compounds. Moreover, a preliminary evaluation of its possible sources revealed that human activities also play an important role in the concentration of OPs in sediments. In 2018, Lee et al. confirmed that the concentration of 18 OPFRs in the sediment of artificial Lake Shihwa were the highest in the world, of which TDCPP and TCPP were dominant in the sediment samples [52]. This paper presented a summary of OPFRs in water and sediment including the most OPFRs measured in previous studies. The detection and data collection of OPFRs concentrations in water and sediment measured were most abundant in previous studies, thus we summarized the results of the predecessors (Table 3) [16,27,32,52,136,137,140,143,144,145,146].

#### 3.3.6. Detection of Organophosphate Flame-Retardant Content in the Atmosphere, Indoor Air and Dust

In the Great Lakes region, the concentration of OPEs in the air was found to be ~100 to ~1200-fold greater than those of brominated flame retardants [147,148]. In 2012, Ali et al. measured the concentration of ten major OPFRs in indoor dust from New Zealand households [39]. The dust samples were taken from the floor of 34 living rooms and 16 mattresses from the same house. The detection result showed that the average concentration of OPFRs were as follows: TEP was 122.5 ng/g, TnBP was 3782.5 ng/g, TCEP was 3812.5 ng/g, TCPP was 3817.5 ng/g, TBEP was 13,687.5 ng/g, TDCPP was 8290 ng/g, TPHP was 17,605 ng/g, and TCP was 1905 ng/g. The concentration of OPFRs in indoor air and dust was measured in 25 unmanned vehicles in Japan in 2017 [149]. The average concentration of OPFRs in the car was 346.5 ng/g, and of which the highest concentrations of TCIPP and TEHP were 390 and 640 ng/g; in the same year, Zhou et al. measured the concentrations of nine OPFRs in 56 indoor and nine outdoor air samples in the main areas of the Rhineland [150], where indoor samples were collected from seven different indoor microenvironments, including private cars, private residences, floor/carpet stores, building materials markets, schools, offices and day-care centres, while outdoor samples were also collected at close proximity to the indoor sampling points. The results showed that the average concentration of OPFRs in the indoor air was 377.15 ng/m^3^. At the same time, TCPP, tris(isobutyl) phosphate (TIBP) and TnBP are the main compounds found both indoors and outdoors. In 2017, Joyce et al. used gas chromatography mass spectrometry and ESI to measure the dust of ten OPFRs in different parts of Brazil, for example, in houses, apartments, offices, primary schools and automobiles the median concentrations of TBOEP (15,900, 22,100, 72,800, 551,000, 62,200 ng/g), TPHP (3900, 3830, 6420, 2210, 86,200 ng/g), TDCIPP (1370, 2250, 4480, 397, 506,000 ng/g) and TCIPP (771, 1870, 1820, 385, 2420 ng/g) were significantly one to three orders of magnitudes higher than the concentration of the other detected OPFRs [151].

## 4. Environmental Impacts of OPFRs

### 4.1. Toxicity

OPFRs are structurally similar to neurotoxic organophosphate pesticides, raising concerns about their exposure and toxicity to humans. Most of the studies on the exposure and toxicity of OPFRs are based on their similarity to organophosphate pesticides.

#### 4.1.1. Developmental Toxicity and Reproduction Toxicity

Developmental toxicity refers to the ability of certain compounds to interfere with the translation and expression function of nucleic acids, thus affecting the growth and development of individuals. The concrete manifestations of developmental toxicity include individual growth retardation, insufficiency or abnormality, teratogenicity and embryonic lethal effect. The insufficiency or abnormality mainly refers to the defects or abnormalities of biochemistry, physiology, metabolism, immunity, nerve activity and behaviour of immature embryos.

Du first studied the developmental toxicity of nine commonly used OPFRs including TEP, TCEP, tripropyl phosphate (TPrP), TCPP, TDCPP, TBEP, TBP, cresyl diphenyl phosphate (CDP) and TPHP with zebra fish embryos, in which were observed embryonic death and malformations, especially pericardial edema. In addition, the effects of alkyl-OPFRs (TPHP and CDP) on cardiac development were also explored by testing the heart rate of zebra fish embryos and the expression of core regulatory genes in cardiac development to explore the mechanism of TPHP and CDP on cardiac development [1]. The results indicated that TPHP and CDP induce cardiotoxicity and impair DNA by affecting the expression of transcriptional regulators. By comparing the LC50 and EC50 (pericardium edema) data, two aryl-OPFRs, TPHP and CDP showed greater heart developmental toxicity than the other OPFRs. It was found that the acute toxicity of OPFRs was primarily dependent on their hydrophobicity. In addition, it was suggested in the study that attention must be paid to pregnant women exposed to OPFRs and their foetal health risks. The study also designed an experiment to compare the cardiac developmental toxicity of a series of aryl-OPFRs and alkyl-OPFRs. The results showed that aryl-OPFRs have greater cardiac developmental toxicity than alkyl-OPFRs. The study also explored possible internal mechanisms relating to this result.

The study by Oliveri explored the possibility that developmental exposure to two OPFRs, TPHP, and TDCIPP. The zebra fish fertilized 0–5 days before were exposed to the TPHP and TDCIPP environments. After the generation of the new-born zebra fish, a battery of functional behaviours was continuously measured from larval to adult zebra fish, including new environment exploration, startle habituation, social attribution and predator escape. The results showed that developmental exposure altered zebra fish behaviour across the lifespan in the above aspects. That is, early developmental exposure to OPFRs produced behavioural impairment that persisted into adulthood as has been observed with the organophosphate pesticides [152].

There were studies that found the developmental exposure of female zebra fish to TDCIPP can lead to decreased levels of dopamine and serotonin in later life [14]. Not limited to newborn fish, Thomas [50] found that OPFRs were also 100% detected in metabolite samples from infants and young children subjects. Compared with adults, faster and more effective nutrient absorption makes it easier for young children to be vulnerable to OPFRs exposure [153]. Additionally, young children are undergoing sensitive periods of metabolism, epigenetic inheritance, neurodevelopment and organ development, which may be disrupted by OPFR exposure [154]. For its toxicity, Thomas assessed the risk of exposure to OPFRs for infant health.

Recently, Meeker and Stapleton suggested that TDCPP and TPHP might be associated with disrupted hormone levels and decreased semen quality among adult human males. Each interquartile range (IQR) increase of TPHP in house dust was associated with a 19% decrease in sperm concentrations and a 10% increase in prolactin level of the adult males [31].

#### 4.1.2. Neurotoxicity

The OPFRs can cause neurodevelopmental toxic effects that are the same as organophosphate pesticides due to the structural similarity between OPFRs and organophosphate pesticides such as chlorpyrifos (CPF), as these organophosphate pesticides have been extensively studied and have been shown to be neurobehavioral teratogens [9,14]. For example, TDCPP exhibits concentration-dependent neurotoxicity in PC12 cells, inhibits DNA synthesis, reduces cell number and alters neural differentiation, which is similar to CPF [9]. Organophosphate-induced delayed polyneuropathy (OPIDP) is a relatively rare neurodegenerative disorder in humans characterized by loss of function, ataxia and paralysis of distal parts of sensory and motor axons in peripheral nerves and ascending and descending tracts of spinal cord, appearing 2–3 weeks after exposure or later [155]. There have been thousands of cases of OPIDP due to triorthocresyl phosphate (TOCP) poisoning in the USA, Morocco, Italy, Romania, Sri Lanka, Yugoslavia and China [156].

Previous studies have reported that the acute neurotoxicity of organophosphate pesticides occurs primarily by inhibiting the action of various forms of cholinesterase, such as acetylcholinesterase (AchE) [157]. Therefore, cholinergic markers (especially AChE activity) have been widely used as biomarkers for determining the neurotoxicity of organophosphates. However, some studies have also reported that the chronic neurotoxic mechanism of organophosphates cannot be related only to cholinesterase inhibition. Therefore, the molecular mechanism behind their toxicity is still elusive [158].

The study by Gu in 2018 investigated the molecular mechanisms of the developmental neurotoxicity for OPFRs by identifying potential targets of OPFRs and the attendant effects [159]. Twelve OPFRs with different substituent groups were evaluated for inhibition of O-GlcNAc transferase (OGT) activity conducting by an electrochemical biosensor. The result showed that the alkyl-OPFRs had no inhibitory effect on OGT. Instead, the six aryl-OPFRs or chlorinated alkyl-OPFRs inhibited OGT activity significantly. Among them, the inhibitory effect of TCrP on OGT was the strongest. Inhibition of OGT by OPFRs might be associated with subsequent toxic effects, including intracellular reactive oxygen species (ROS), calcium levels, and cell proliferation and autophagy. Molecular docking of the OGT/OPFR complexes was carried out to reveal the rationales of difference in their structure-dependent inhibition potency.

### 4.2. Biological Enrichment

Numerous works showed high concentrations of OPFRs in comparison to the average levels found for PBDEs in certain organisms, which indicates that OPFRs could be somehow bioaccumulated, even though their *K*_ow_ values are not as large as those of PBDEs or other persistent organic pollutants [125].

Octanol-water partition coefficients (*K*_ow_) can simulate the distribution of organics between a biological phase and the water phase, which is closely related to the toxicity, biological enrichment and solubility of compounds. OPFRs possess positive log*K*_ow_ values (range 0.8–10.6), which is indicative of their potential for bioaccumulation in biota [4].

14 OPFRs were analysed in egg pools of 10–13 individual herring gull eggs from five colonial nesting sites for 11 years spanning 1990–2010 in the Laurentian Great Lakes of North America (Chantry Island, Fighting Island, Agawa Rocks, Toronto Harbour and Gull Island) [160]. The concentrations of OPFRs in crucian carp were calculated by wet weight-based units, as it has already been demonstrated that most OPFRs are not dependent on lipid content despite their lipophilic characteristics [24]. In the plasma of herring gulls from Lake Huron, Su et al. reported the detection of the OPFR diester metabolites bis (1,3-dichloro-2-propyl) phosphate (BDCIPP), bis-(2-butoxyethyl) phosphate (BBOEP) and di(2-ethylhexyl) phosphate (DEHP) [161].

Recently, two studies were conducted to assess the effects of various factors such as trophic levels and fish size on the bioaccumulation of OPFRs in diverse fish species [24,162]. They found no correlations between the concentrations of OPFRs and trophic levels through the food web or lipid contents, and no growth-dependent accumulation of OPFRs in any fish species.

In addition to the bioaccumulation of OPFRs found in seagulls and various kinds of fish, the metabolites of OPFRs have also been detected in humans, indicating that humans are not only exposed to OPFRs by ingestion and inhalation but also metabolize OPFRs in vivo.

Thomas investigated the demographic and dietary survey data in reference to OPFR urinary metabolite concentrations in 15-to-18-month-old infants, OPFR metabolites were detected in all samples. The highest detection rate was the metabolite of TPHP, diphenyl phosphate (DPP), detected in 100% of subjects, followed by the metabolite of TDCPP, 85–95% for BDCIPP, and isopropylated triphenyl phosphate (ITP) metabolite, 81% for mono isopropylphenyl phenyl phosphate (ip-DPP). In addition, it was revealed that meat and fish consumption may be associated with higher levels of the OPFRs metabolites DPP and BDCIPP [50].

Through Choo found there were no correlations between the concentrations of OPFRs and trophic levels in any fish species, that is, the concentration level of OPFRs does not increasingly accumulate with an increase in trophic level in the food net. However, human beings are the most advanced consumers at the top of the food web. It can be speculated that in addition to OPFRs in the exposure pathways such as inhalation and skin absorption, humans can also enrich OPFRs in the body by eating OPFRs-rich animals or fish [163].

Most of these studies investigated the edible parts (muscle) of fish to evaluate the presence and risk assessment of OPFRs [24,164]. At present, the new research ideas are to explore the tissue-specific distribution of OPFRs in vivo.

Choo first simultaneously investigated the fate (concentrations, distributions, and bioaccumulation) of OPFRs in biotic and abiotic media and to show sex differences [163]. The study showed the highest concentrations were observed in liver (6.22–18.1 ng/g ww), and the levels in muscle (4.23–7.75 ng/g ww) and gonad (3.08–7.70 ng/g ww) in biotic media, and OPFR concentrations in abiotic media river water and sediment were 62.8–961 ng/L and 2.70–7.43 ng/g dw, respectively. Since the liver is the first tissue perfused by trace pollutants through blood flow and has a higher assimilation rate than muscle [165,166]; one study identified different accumulation characteristics of OPFRs in several organs of freshwater fishes, observing the highest concentrations in liver, followed by kidney, muscle, intestine, and ovarian tissues [167].

Ma et al. recently reported OPFR concentrations in muscle tissue of domestic chickens (Gallus gallus domesticus) and ducks (Anas platyrhynchos domesticus). TnBP, TCEP, TBOEP, and TPHP were present at the highest concentrations (up to 281 ng/g lipid weight; 14 ng/g wet weight) [129].

Greaves and Letcher reported on the 16 OPFRs body compartment composition and in ovo or in utero transfer, that is, concentrations and distribution of triesters in eight tissues from female herring gulls (Larus argentatus) and their entire clutches of eggs in 2010 from a Lake Huron colony site, Laurentian Great Lakes of North America. In all measured samples, fat (32.3 ± 9.8 ng/g wet weight; ww) contained the highest ΣOPFRs concentration followed by egg yolk (14.8 ± 2.4 ng/g ww) ≈ egg albumen (14.8 ± 5.9 ng/g ww) > muscle (10.9 ± 5.1 ng/g ww) ≫ red blood cells (1.00 ± 0.62 ng/g ww). In contrast, in liver, blood plasma, and brain all OPFRs were not detectable. Six different tissues in the female herring gulls (Larus argentatus) at the Huron colony site, and the concentration of 16 OPFRs in the egg yolk and protein of their eggs were determined. In the fat, egg yolk, muscle, and blood cells of the gull, the presence of OPFRs was detected, while in the liver, plasma, and brain, no OPFRs were detected, and the concentration and ratio of the nine OPFRs present depended on the body and egg compartment [3].

### 4.3. Endocrine Disrupting Effects

Exposure to OPFRs has been proved to be associated with many adverse effects. Alteration in thyroid function and change in relative liver weight were also reported in laboratory animals. Various studies have confirmed that the accumulation of OPFRs in vivo is indeed associated with endocrine disruption mechanisms.

Liu used some kinds of cell lines of human and zebra fish to study the endocrine disrupting potential of six OPFRs (TCEP, TCPP, TDCPP, TBEP, TPHP, tricresyl phosphate (TCP)), and TCP by using the oestrogen receptor binding activity of OPFRs as an indicator of endocrine disruption. The results of this study showed that the above six OPFRs compounds could alter the sex hormone balance through several mechanisms including alterations of steroidogenesis or oestrogen metabolism [45].

In the study of Kojima, the authors characterized the agonistic and antagonistic activities of 11 OPFRs against human nuclear receptors using cell-based transactivation assays; the results suggest that several OPFRs may have potential endocrine disrupting effects via oestrogen receptor, androgen receptor, glucocorticoid receptor and pregnane X receptor. Additionally, in the later research, the author investigated the effects of primary metabolites of organophosphate flame retardants on transcriptional activity via human nuclear receptors, this time, the agonistic and/or antagonistic activities of 12 primary OPFR-metabolites against ten human nuclear receptors were examined using the same methods as before and compared to those of their parent compounds. The results suggest that some of primary metabolites such as hydroxylated TPHP-metabolites show increased oestrogenicity compared to the parent compound, whereas the others (for instance diester OPFR-metabolites) may have limited nuclear receptor activity compared with their parent triester OPFRs [167].

The above studies revealed that OPFRs scattered in various media can not only directly interfere with the human endocrine system but also the metabolites produced by metabolism in the body will have endocrine disrupting effects on the human body.

## 5. Pollution Controls of OPFRs

In view of the hazards of OPFRs exposure to the environment and organisms, it is particularly important to find reasonable ways to remove OPFRs from the environment. At present, the main methods for removing OPFRs are the activated sludge method, biofilm method, photoconversion method, adsorption method and so on.

For FRs, there are many researches describing thermal treatment methods to remove them [168,169,170,171], high temperatures technologies include incineration (waste-to-energy), pyrolysis, gasification, plasma treatment, and supercritical water oxidation (SCWO) were used to destroy and remove FRs [172,173]. However, the amount and type of FRs may alter the removal processes, thus FRs cannot simply be added to the processes, and it has turned out that converting demonstration and laboratory-scale units into industrial systems is difficult [169]. In addition, halogenated FRs introduce the potential for toxic byproducts, including halogenated dioxins and furans and thermal treatment may reduce the need for reuse and recycling [174]. Therefore, the thermal treatment methods for removing FRs are not an optimal option.

Using the sludge method to remove the OPFRs, the target contaminant remaining in the activated sludge is difficult to degrade and easily re-enters the environment by bioaccumulation. Cristale et al. found that the conventional activated sludge method could not effectively remove OPFRs [5], and the research studied the removal efficiency of OPFRs by activated sludge and advanced oxidation process (AOP) from wastewater from five WWTPs in Catalonia, Spain, and proposed to apply ozonation and UV/H_2_O_2_ to the actual sewage treatment plant treatment process. Although it was proved that alkyl-OPFR (TBOEP, TnBP and TIBP) can be effectively removed through O_3_ and UV/H_2_O_2_, chlorinated (TCEP, TCIPP and TDCPP) compounds were not degraded, that is, the improved AOP can remove some OPFRs, but the remaining OPFRs still produce great harm to the environment; Deng et al. found that microfiltration/reverse osmosis is essential for the removal of OPFRs and heavy metals through the treatment of OPFRs and heavy metals in the landfill leachate treatment system in Guangzhou, while core biological treatments have a small effect on the removal and only played a secondary role [175]. Krzeminski et al. used ultrafiltration (UF), nanofiltration (NF) and reverse osmosis (RO) membranes for membrane filtration to remove OPFRs from municipal wastewater, improving the quality of municipal wastewater to be conducive to the reuse of urban sewage [176]. However, after filtration, the OPFRs still remain a source of pollution; its final treatment is still a problem. Due to their refractory organics and bioaccumulation, they easily re-enter the environment causing re-contamination, thus the final degradation treatment of OPFRs is particularly important.

Cristale et al. used phototransformation to remove OPFRs, analysing whether light transformation of alkyl, chloroalkyl and aryl-OPFRs causes photoconversion in river water [151]. The results showed that as an important method for removing OPFRs, the method can convert the macromolecular OPFRs organics into smaller molecular substances, thereby completely solving the problem of OPFRs exposure in the environment, but it only had effects on the phototransformation of some kinds of OPFRs. The study also pointed out that it was necessary to further study the role of certain OPFRs as photosensitizers. Furthermore, there are many photoconversion products generated during the photolysis process for numerous OPFRs compounds even in some cases the byproducts can be more toxic than the original substance.

Wang et al. first studied the removal of OPFRs by activated carbon. The adsorption kinetics and characteristics of activated carbon, properties of OPFRs and the pH value of the solution, as well as the adsorption mechanism and reuse were studied in this research [177]. However, the final treatment of the OPFRs after adsorption is still a difficult problem to solve.

The abovementioned method has certain effects on removing certain kinds of OPFRs, although its cost was high, it required considerable manpower and material resources, the treatment cycle was long, and the removal was not thorough enough; also, few OPFR substances can be best removed by this method. Moreover, in the process of removing organophosphate flame retardants, a series of by-products may also cause secondary damage to the environment. Therefore, it is important to look for alternatives to OPFRs.

Quantitative structure-activity relationship (QSAR) is a comprehensive quantum chemical calculation and mathematical statistical method for quantitatively studying the correlation between compound structure and activity. This method plays a key role in drug design, such as drug discovery, optimization, absorption, metabolism and toxicity aspects.

The purpose of QSAR research mainly includes the following three aspects: (1) exploring the factors affecting the biological activity of the compounds, (2) improving the known lead compounds and increasing their activity, and (3) predicting the activity of compounds with unknown activity [178].

Wang et al. conducted a molecular structure design of pentachlorophenol (PCP) bioconcentration by 3D-QSAR, and finally designed a bioaccumulation reduction (reduced by 32.89%) with the insecticidal effect basically unchanged (increased by 1.37%) and organized an environmentally friendly 6-COCl-PCP molecule with increased degradability (24.81% increase) and substantially no migration (0.94% reduction) [179]. Zhao et al. completed a molecular design of polychlorinated naphthalene based on 3D-QSAR and fractional factorial design, designing 34 kinds of novel molecules with reduced bioaccumulation, and calculated Gaussian 09 for 34 new molecules [180]. The results showed 34 species. Compared with the target molecule, the novel CN-42 molecule has reduced bioconcentration and its stability, insulation and flame retardancy remain basically unchanged. However, the toxicity and long-range mobility of some molecules are decreased. Chu et al. constructed a molecular modification scheme of common PBDEs in flame retardants for low oestrogen-activity PBDEs by using the 2^10-3^ fractional factorial experimental design method with resolution V and the pharmacophore model [181]. A total of 40 new low oestrogenic-activity PBDEs were designed, and the flame retardancy of the newly designed PBDEs remained unchanged. At the same time, the biological toxicity, environmental persistence, long-distance migration and bioaccumulation decreased to varying degrees. This study can provide theoretical guidance for the design of low oestrogen-activity and environmentally friendly alternatives to PBDEs.

Using molecular design to find environmentally friendly alternatives that meanwhile maintain the intrinsic practical functional can not only solve its environmental pollution problems at the source but also fully retain their functional characteristic, which is a new research direction for solutions to the current emerging pollutants, OPFRs.

## 6. Conclusions

In summary, this study has attempted to give a comprehensive review of the latest developments of emerging FR OPFRs, expound the environmental hazards and impacts of OPFRs on the health of organisms, and systematically introduce the classification, application, typical synthetic routes, occurrence and distribution in various environments as well as the testing methods of OPFRs. In addition, the bioaccumulation of OPFRs (in marine or river aquatic organisms and humans), toxicity (developmental toxicity, reproductive toxicity, and neurotoxicity), and related environmental pollution control are comprehensively reviewed.

Through the process of review, it was found that the concentration levels of different kinds of OPFRs in non-biological media may be different due to their different molecular structural characteristics (hydrophilicity or hydrophobicity). The reason for their tissue specificity in animal and human enrichment is also because of its structural characteristics. Most of the current research can only obtain a qualitative conclusion, but research about the internal mechanisms of OPFRs enrichment in biological and non-biological media is still relatively scarce.

In addition, as emerging pollutants, OPFRs have experienced less research on environmental conversion, that is, the removal, and the removal effect of OPFRs is barely satisfactory. At present, there is still no way to effectively degrade OPFRs to reduce their environmental hazards. The reasons for its recalcitrance to degradation are also not clear. This paper thus proposes to seek environmentally friendly alternatives for OPFRs considering both the donor and the receptor by decreasing the enrichment of OPFRs in a variety of media using modification of the molecular structure of OPFRs or altering the structure of the receptors bound to OPFRs in the environment. In other words, by modifying the existing OPFRs at the source, some environmentally friendly alternatives such as the theoretical substitution for low-toxicity OPFRs molecular modification and generation could be applied in order to provide new ideas and theoretical guidance for the removal of OPFRs. In general, this paper hopes to provide new ideas and theoretical guidance for the removal of OPFRs.

## Figures and Tables

**Table 1:** Restrictive regulation for flame retardants.

Type	Compounds
POPs	Decabromodiphenyl ether (commercial mixture, c-decaBDE)
	Hexabromocyclododecane
	Hexabromobiphenyl
	Hexabromodiphenyl ether
	Heptabromodiphenyl ether
	Commercial octabromodiphenyl ether
	Short-chain chlorinated paraffins
	Tetrabromodiphenyl ether
	Pentabromodiphenyl ether
REACH	Short-chain chlorinated paraffins
	Tris(2-chloroethyl) phosphate
	Boric acid
	Sodium tetraborate
	Hexabromocyclododecane
	Tris-(2-carboxyethyl)-phosphine hydrochloride
	Decabromodiphenyl ether
	Boron oxide

**Table 2:** Comparison of organophosphate flame retardants (OPFRs) with other flame retardants (FRs).

Name	Category	Molecule Structure	Solubility (mg/L) at 25 °C	VP (mmHG) at 25 °C	log*K*_ow_	BCFs	Ref.
Tricresyl phosphate	OPFRs	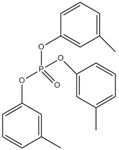	0.36	1.80 × 10^−7^	5.48	8.56 × 10^3^	[2,4,16]
Tributyl phosphate		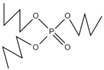	280	1.13 × 10^−3^	4.00	39.81	
Tri-iso-butyl phosphate		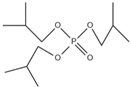	475.57	0.0129	3.60	19.51	
Triethyl phosphate		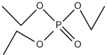	1115	0.393	0.87	3.162	
Tris(2-ethylhexyl) phosphate		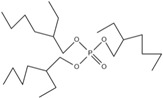	0.6	8.25 × 10^−8^	9.49	1 × 10^6^	
Triphenyl phosphate		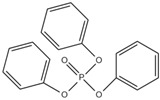	1.9	1.12 × 10^−5^	4.70	113.3	
Tris(2-chloroethyl) phosphate		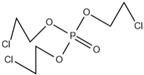	7000	0.0613	1.63	0.4254	
Tris(chloroisopropyl) phosphate		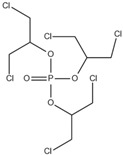	51.85	5.64 × 10^−5^	2.89	3.268	
Tris(2-butoxyethyl) phosphate		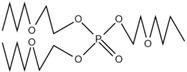	1100	1.23 × 10^−6^	3.00	25.56	
Trixylenyl phosphate		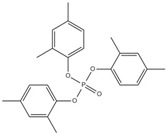	0.89	8.76 × 10^−8^	7.98	480.1	
2-ethylhexyldiphenyl phosphate		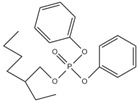	1.9	5 × 10^−5^	6.30	855.3	
Resorcinol bis(diphenyl phosphate) (RDP)		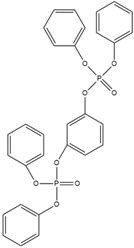	1.11 × 10^−4^	2.1 × 10^−8^	5.82	2.05 × 10^4^	
Decabromodiphenylethane	BFRs	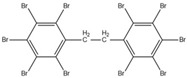	2.1 × 10^−7^ g/Lb	333–349	11.1		[70,110]
1,2-Bis(2,4,6-tribromophenoxy)ethane		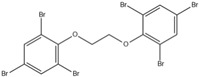	0.72 μg/L	350	3.55		
2-Ethylhexyl-2,3,4,5-tetrabromobenzoate		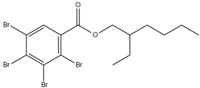	3.70 × 10^−5^ mg/L		8.26		
pentabromodiphenyl ether (PentaBDE)	PBDEs	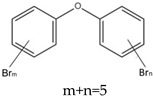	13.3	2.2 × 10^−7^ to 5.5 × 10^−7^	6.64 to 6.97		[63,81]
octabromodiphenyl ethers (OctaBDE)		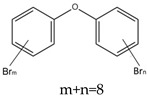	Less than 1	9.0 × 10^−10^ to 1.7 × 10^−9^	6.29		
decabromodiphenyl ether (DecaBDE)		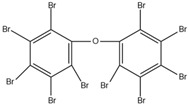	Less than 1	3.2 × 10^−8^	6.265		
Hexabromocyclododecane (HBCDD)	NFRs	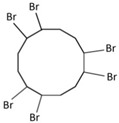					[111]
Tetrabromobisphenol A (TBBPA)		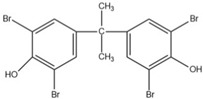	Insoluble (<1 mg/mL) (NTP, 1992)	1.37 × 10^−8^	5.90		[70]

**Table 3:** Summary of mean (min-max) concentrations of OPFRs in water and sediment measured in previous studies (ng/L).

Country	Sample Year	TBP	TCEP	TCPP	TDCPP	TBEP	TPhP	TEHP	ΣOPFR	Reference
Great Lakes	2011–2012	1.03	0.72	5.87	1.99	27.6	0.49			[143]
Elbe River, Germany	2013	41.0 (5.2–108)	75.9(33.8–104)	104(89.9–128)	76.7(51.8–111)					[144]
Nalón, Arga and Besòs River, Spain	2012	92.1(<LOQc–370)	85.3(<LOQ–330)	571(<LOQ–1800)	103(<LOQ–200)	1445(<LOQ–4600)	11.6(<LOQ–35.0)	2.01(<LOQ–4.00)	1072(<LOQ–7200)	[32]
German Bight (North Sea), Germany		33.7(<LOQc–84)	22.9(3.29–69.9)	146(24.3–570)	22.5(5.30–67.0)	50.7(<LOQ–103)	5.90(<LOQ–10.3)		402(58.3–1092)	[27]
North China	2013	13.4(<LOQc–218)	80.2(1.30–268)	186(4.60–921)	4.30(<LOQ–44.0)	4.20(<LOQ–47.0)	1.00(<LOQ–15.7)	0.39(<LOQ–3.30)	398(9.55–1550)	[145]
Qingdao, Lianyungang and Xiamen, China			134(21.0–618)	84.1(15.8–170)	109(24.0–378)				425(91.9–1392)	[137]
Lake Shihwa, Korea	2015	24.8(<LOQc–72.9)	255(3.26–5963)	211(<LOQ–5100)	15.6(<LOQ–325)	164(2.88–838)	8.29(<LOQ–96.2)	3.27(<LOQ–59.4)	877(28.3–16,000)	[52]
Sediment (ng/g dry weight) Great Lakes	2010–2013	0.58(<LOQc–1.96)	1.40(<LOQ–1.90)	0.46(<LOQ–3.37)	0.70(<LOQ–1.99)	3.54(<LOQ–23.7)	1.17(<LOQ–9.03)	0.41(<LOQ–8.38)	9.01(0.44–48.0)	[140]
Nalón, Arga and Besòs River, Spain	2012	6.82(<LOQc–13)	6.02(<LOQ–9.70)	116(<LOQ–365)	7.96(<LOQ–12.0)		7.41(<LOQ–23.0)	35.1(<LOQ–290)	156(3.80–824)	[32]
Evrotas River Basin, Greece	2014–2015	2.39(<LOQc–5.54)	1.76(<LOQ–2.27)	4.59(<LOQ–7.62)	1.63(<LOQ–2.96)	1.47(<LOQ–3.35)	0.36(<LOQ–0.67)	1.84(<LOQ–4.73)	10.4(0.31–31.0)	[136]
Adige River Basin, Italy	2014–2015	5.52(<LOQc–42.6)	2.52(0.33–19.0)	14.9(0.53–53.7)	2.31(<LOQ–6.86)	2.36(<LOQ–9.98)	1.49(<LOQ–9.69)	8.97(<LOQ–35.1)	82.6(11.5–549)	[136]
Sava River Basin, Serbia	2014–2015	7.65(<LOQc–14.2)	0.79(<LOQ–2.32)	6.60(<LOQ–14.7)	0.36(<LOQ–0.39)	3.16(<LOQ–11.0)	<LOQ	3.53(0.33–7.73)	50.1(10.5–248)	[136]
Taihu Lake, China		1.04(<LOQc–2.65)	1.75(0.62–3.17)	1.36(<LOQ–2.27)	1.16(<LOQ–5.54)	2.00(1.03–5.00)	0.49(<LOQ–1.19)		7.88(3.38–14.3)	[16]
Pearl River Delta, China	2015	3.53(1.29–12.8)	7.36(1.00–26.5)	18.3(2.27–186)	1.31(<LOQ–6.05)	4.08(<LOQ–19.7)	14.3(0.42–317)	3.74(0.15–26.7)		[137]
Pearl River Delta, China	2010	7.11(<LOQc–37)	13.1(<LOQ–58.0)	15.7(0.91–185)	1.59(<LOQ–10.0)	10.4(<LOQ–46.0)	26.9(<LOQ–253)	13.1(0.86–56.0)	78.3(8.31–470)	[146]
Lake Shihwa, Korea	2015	2.14(<LOQc–33.3)	18.4(<LOQ–290)	194(<LOQ–2500)	43.6(<LOQ–405)	64.4(<LOQ–2755)	18.7(<LOQ–257)	3.49(<LOQ–99.7)	381(3.00–3800)	[52]

## Abbreviations

OPFRs	organophosphate flame retardants
ECs	emerging contaminants
FRs	flame retardants
ATH	alumina trihydrate
VP	vapour pressure
BCF	bioconcentration factor
TBP	tributylphosphat
TEP	triethyl phosphate
TCEP	tri(2-chloroethyl)phosphate
BFR	brominated flame retardant
PBDE	polybrominated diphenyl ether
PentaBDE	pentabromodiphenyl ether
OctaBDE	octabromodiphenyl ethers
DecaBDE	decabromodiphenyl ether
NBFR	new brominated flame retardant
AFR	alternative flame retardant
WWTP	wastewater treatment plant
TBOEP	tris(2-butoxyethyl) phosphate
TCIPP	tris(1-chloro-2-propyl) phosphate
PU	polyurethane
ABS	acrylonitrile-butadiene-styrene
HIPS	high impact polystyrene
PVC	polyethylene, polyvinyl chloride
TCPP	tris(chloroisopropyl) phosphate
TDCPP	tris(1,3-dichloroisopropyl) phosphate
TDCIPP	tris(1,3-dichloroisopropyl) phosphate
TH	thyroid hormone
DCRP	DechloranePlus
POPs	persistent organic pollutants
REACH	Registration, Evaluation, Authorization and Restriction of Chemicals
RDP	Resorcinol bis(diphenyl phosphate)
DBDPO	decabromodiphenyl oxide
DCEPP	O,O′-di (2-chloroethyl),O″-[2-bis-(2-chloroethoxy) phosphoryl] propylphosphate
DBEPP	O,O′-di (2-bromoethyl),O″-[2-bis-(2-bromoethoxy) phosphoryl] propylphosphate
LOI	limiting oxygen index
PMPC	phenyl methyl phosphonochloridate
MPDC	methyl phosphonic dichloride
DPPMP	diphenyl piperazine-1,4-diylbis(methylphosphinate)
PSF	polysulfone
BMI	bismaleimide
DGEBA	diglycidyl ether of bisphenol A
HNCP	hexakis (4-nitrophenoxy) cyclotriphosphazene
PET	poly(ethylene terephthalate)
POBPP	polyphosphate poly (6-oxido-6H-dibenzo [-c,e] [1,2]oxa-phosphinin-6-yl phenyl phenylphosphate
DOPO-BQ	10-(2,5-dihydroxyl phenyl)-9,10-dihydro-9-oxa-10-phosphaphenanthrene-10-oxide
HBCDD	hexabromocyclododecane
TBBPA	tetrabromobisphenol A
LC/MS	liquid chromatography-mass spectrometry
TPHP	TPHP (triphenyl phosphate)
BDCP	BDCP (Tris-(2,4,6-Tribromophenoxy)dichlorophosphorane)
TBEP	TBEP (tris(2-butoxyethyl) phosphate)
HPLC	high performance liquid chromatography
LC-MS-MS	liquid chromatography-quadrupole-linear ion trap mass spectrometry
LC-ESI/MS/MS	liquid chromatography–electrospray ionization (+)– tandem mass spectrometry
MS/MS	mass spectrometer
ESI	electrospray ionization
SRM	selected reaction monitoring
MAE	microwave assisted extraction
OPE	organophosphate ester
IPPP	tris(isopropylphenyl)phosphate
IDPP	isodecyldiphenyl phosphate
EHDP	ethylhexyl diphenyl phosphate
TEHP	tri(2-ethyltexyl)phosphate
TnBP	tri-n-butyl phosphate
TDBPP	tris(2,3-dibromopropyl) phosphate
TDCP	tris(1,3-dichloropropyl)phosphate
MQL	method quantification limits
PRE	pearl river estuary
TMPP	tris(methylphenyl) phosphate
TCrP	tricresyl phosphate
TPP	tritolyl phosphate
TIBP	tris(isobutyl) phosphate
TPrP	tripropyl phosphate
CDP	cresyl diphenyl phosphate
IQR	interquartile range
CPF	chlorpyrifos
OPIDP	organophosphate-induced delayed polyneuropathy
TOCP	triorthocresyl phosphate
AchE	acetylcholinesterase
OGT	O-GlcNAc transferase
ROS	reactive oxygen species
BDCIPP	diester metabolites bis (1,3-dichloro-2-propyl) phosphate
BBOEP	bis-(2-butoxyethyl) phosphate
DEHP	di(2-ethylhexyl) phosphate
DPP	diphenyl phosphate
ITP	isopropylated triphenyl phosphate
TCP	tricresyl phosphate
AOP	advanced oxidation process
UF	ultrafiltration
NF	nanofiltration
PCP	pentachlorophenol
SCWO	supercritical water oxidation
CPSC	consumer products safety commission
